# Bioactive Compounds from Vine Shoots, Grape Stalks, and Wine Lees: Their Potential Use in Agro-Food Chains

**DOI:** 10.3390/foods10020342

**Published:** 2021-02-05

**Authors:** Marica Troilo, Graziana Difonzo, Vito M. Paradiso, Carmine Summo, Francesco Caponio

**Affiliations:** 1 Department of Soil, Plant and Food Sciences, Food Science and Technology Unit, University of Bari Aldo Moro, Via Amendola, 165/A, I-70126 Bari, Italy; marica.troilo@uniba.it (M.T.); graziana.difonzo@uniba.it (G.D.); carmine.summo@uniba.it (C.S.); 2 Department of Biological and Environmental Sciences and Technologies, University of Salento, S.P. 6, Lecce-Monteroni, I-73100 Lecce, Italy; vito.paradiso@unisalento.it

**Keywords:** winery by-products, bioactive compounds, dietary fiber, polyphenols, natural additives

## Abstract

The winemaking sector is one of the most productive worldwide, and thus it also generates large amounts of by-products with high environmental impacts. Furthermore, global market trends and government regulations promote industrial alternatives based on sustainable production processes. As a result, several studies have focused their attention on the reuse of grape by-products in the agro-food chain. Vine shoots, grape stalks, and wine lees, although produced to a lesser extent than grape pomace, have increasingly been receiving attention for their applications in the food sector, since they are a good source of functional and bioactive compounds. In this framework, our review highlights the promising results obtained by exploiting the antioxidant and/or antimicrobial activity of vine shoots, grape stalks, and wine lees or their extracts to replace the most common oenological additives and to assay the activity against food pathogens. Further, innovative functional foods and sustainable food packaging have been formulated by taking advantage of polyphenols and fiber, as well as plant bio-stimulants, in order to obtain grapes and wines with high quality characteristics. Overall, these by-products showed the potential to be recycled into the food chain as functional additives for different products and applications, supporting the sustainability of the winemaking sector.

## 1. Introduction

Grape is one of the largest fruit crops in the world, and according to the International Organization of Vine and Wine (OIV), in 2019 the world grape production was of 77.8 million tons, of which 57% was wine grapes, 36% was table grapes, and 7% was destined for the production of dried grapes [1]. Winemaking is one of the main agro-industrial activities in the world, and *Vitis vinifera* L. is the most used for industrial wine production [2,3,4]. According to estimates by the OIV, wine production in 2020 will reach 258 million hectoliters, with Europe as the main wine producer, followed by America and Asia. Among the main wine producers, Italy is the world leader, with production of about 49 million hectoliters, followed by France and Spain [5].

Winemaking produces a large quantity of wastes and by-products in a short period of time [6], corresponding to approximately 30% *w*/*w* of the starting grapes [7,8,9], represented by grape pomace, grape seeds, grape stalks, and wine lees, as well as wastewater [3,6,10,11,12]. These by-products are considered to be highly polluting due to the presence of organic substances, pH, salinity, and heavy metal content, thus having negative repercussions on environmental and economic sustainability [2,9,12,13,14].

Grape pomace is the solid residue obtained following the pressing and the fermentation process [10,15,16] and represents 20–25% (*w*/*w*) of the total weight of the grape used for wine production and consists mainly of skin, pulp, stalk residuals, and seeds [10,11,17,18,19]. Traditionally grape pomace is used to produce feed, fertilizer, or different types of distillates [16,20,21,22,23]. However, in recent years, due to its chemical composition, grape pomace was considered a promising alternative to obtain high value added products [22]. Seeds are rich in antioxidant compounds, such as vitamin E [3] and phenolic compounds, phytosterols, fibers, proteins, carbohydrates, and minerals, but especially in lipids. Grape skin and pulp, instead, are a rich source of fibers and phenolic compounds including protocatechuic, gallic, vanillic, and caftaric acid; flavonols such as quercetin, myricetin, and kaempferol; and anthocyanins, whose presence is closely dependent on the vinification procedures and grape variety considered [24,25,26,27].

As already reported, winemaking also generates grape stalks, following destemming, and wine lees, the residue that forms after wine fermentation at the bottom of recipients. Grape stalks (about 7%, *w*/*w* of grape total weight) are usually removed before the fermentation phase to avoid excessive wine astringency [12,28]; they are a source of phenolic compounds such as tannins, flavan-3-ols, hydroxycinnamic acids, monomeric and oligomeric flavonols, and stilbenes [28,29], and lignocellulosic compounds such as hemicellulose, cellulose, and lignin [2,9,30]. Wine lees (about 5%, *w*/*w* of grape total weight), instead, contain mainly ethanol, tartaric acid, phenolic compounds, and yeast cells [15,23,31].

In addition, the agronomic practice of pruning generates an enormous quantity of agricultural wastes, principally vine shoots, with a production estimated per year of about 1–2 tons per hectare [32]. They are usually shredded and used as a soil conditioner, to improve fertility, or as biomass to produce energy. More recently, they have been used as a source of bioactive compounds (dietary fibers, phenols, proteins, lipids, hydrocolloids) for food, pharmaceutical and cosmetic industries, due to the nutritional benefits [15,23]; while grape leaves, due to their presence of organic acids, lipids, and polyphenols, can be used in the cosmetics industry [12].

Vine shoots, grape stalks, and wine lees, although produced in smaller quantities than grape pomace, represent a rich source of functional compounds. In this framework, the purpose of this review is therefore to highlight the potential use of these wastes and by-products in the food sector as (i) antimicrobial and antioxidant agents; ii) natural additives; (iii) nutritional and food quality improver; and (iv) filler in food packaging formulations.

## 2. Health and Preservative Properties of the Bioactive Compounds

**Figure 1:**
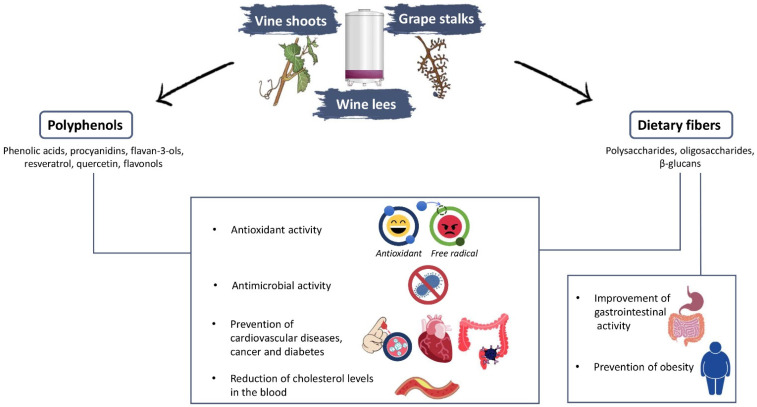
Effects of phenolic compounds and dietary fiber on health.

The increasing attention paid to human health has led researchers towards the recovery and the identification of bioactive compounds in vegetable matrices, such as by-products. These bioactive compounds are mainly represented by polyphenols—secondary metabolites produced by plants under stress conditions and involved in the defense against pathogens, environmental stress, and ultraviolet radiation [3,28]—and by dietary fiber, defined as carbohydrate polymers with 10 or more monomeric units that are not hydrolyzed by the endogenous enzymes in the small intestine of humans [33] (Figure 1).

With regard to winery by-products, four main categories of polyphenols have been identified: (i) phenolic acids, present in the form of hydroxybenzoic and hydroxycinnamic acids [10,21]; (ii) flavonoids, divided into different classes, namely flavones, flavanons, flavonols, isoflavones, anthocyanins, and proanthocyanidine [22]; (iii) tannins; and (iv) stilbenes, mainly represented by trans-resveratrol and ε-viniferina [18,28,34,35]. Numerous studies have highlighted the beneficial effects of phenolic compounds, which exert antimutagenic, anticarcinogenic, anti-inflammatory, and antioxidant activity [18,21]. Specifically, trans-resveratrol, catechin, epicatechin, quercetin, and phenolic acids have been studied for involvement in the prevention of cardiovascular diseases, cancer, diabetes, osteoporosis, and neurodegenerative diseases [28]. In particular, they are involved in the reduction of low-density lipoproteins (LDL) [3,36,37], the consequent increase of high-density lipoproteins (HDL), and the prevention of platelet aggregation [28]. Grape phenolic compounds promote vasodilation [35], reduce urinary F2-isoprostanes and other oxidative stress markers, and increase the serum oxygen radical absorbance capacity, thus decreasing the oxidation of plasma proteins in healthy and sick patients [36].

In addition, an important role for human health is also played by dietary fiber, the recommended consumption of which is about 25–30 g per day [38], which is generally taken as a result of the ingestion of cereals, fruits, and vegetables. Due to modern dietary habits, this value is difficult to achieve; for this reason, it is necessary to take alternative sources of dietary fiber to achieve the recommended daily amount. An adequate fiber intake is associated with the prevention of cardiovascular diseases, hypertension, diabetes, and obesity [21,39,40]. Further, one of the main benefits is the improvement of gastrointestinal activity in terms of motility, by modulating gastrointestinal transit time, the fecal weight, and the fecal acidity [41], followed by satiety promotion and modulation of the immune responses of the intestinal mucosa [18]. Dietary fiber can also reduce glycemic responses and cholesterol levels in the blood [41], delaying and interfering in the absorption of cholesterol and bile acids [21], but also by limiting the carbohydrate absorption, thus reducing insulin response and triacylglycerol levels, which are risk factors for coronary heart disease [39]. Some researchers showed a positive effect, due to the intake of foods containing dietary fiber and polyphenols, on the chemo preventive effects on intestinal tumorigenesis [42,43].

Dietary fiber, as well as polyphenols, can also exert antioxidant and antimicrobial action. In the food sector, microbial activity and lipid oxidation are the first indices of food spoilage, which gives rise to concerns for the food industry and for consumers [44].

Lipid oxidation is one of the main factors linked to oxidative rancidity, the loss of essential fatty acids, and the development of unpleasant flavor and smells [22]. The antioxidant activity of polyphenols is exerted by free radical inactivation, electron transfer, and scavenging and neutralizing reactive oxygen species (ROS), stopping the oxidation propagation phase and preventing the formation of peroxides [22]. They can also delay the oxidation process by acting as metal chelators to convert hydroperoxides or metal pro-oxidants into stable compounds [45,46,47].

The antimicrobial capacity exerted by polyphenols is frequently associated with mechanisms of depolarization and permeability of cell membranes, the decrease of bacterial proteins and loss of cytoplasm, inhibition of extracellular microbial enzymes, direct action on microbial metabolism, saturation of action sites, inhibition of nucleic acid synthesis, and deprivation of substrates necessary for bacterial growth through a chelation mechanism in the presence of metals [28,46,48,49]. Further studies highlighted the involvement of polyphenols and dietary fiber in the inhibition of pathogens such as *Staphylococcus aureus*, *Bacillus cereus*, *Salmonella*, *Escherichia coli* [22,50,51,52,53], and *Listeria innocua* ATCC 51142, demonstrating potential applications to preserve foods and prolong their shelf-life [41].

Besides antimicrobial and antioxidant action, the addition of oenological by-products also seems to influence the qualitative and organoleptic characteristics of fortified products with a significant impact on the phenolic and volatile component, and on color, texture, and sensory characteristics. Many reviews in the literature, in fact, underline the growing interest of researchers in the bioactive compounds present in grape pomace and seed, and their effects not only on human health, but also on food properties [16,18,21,22,41,54].

## 3. Food Applications

The remarkable amount of winemaking sector by-products has led researchers to look for alternative uses to enhance them. In Table 1 are reported the possible applications of grape stalks, vine shoots, and wine lees as food ingredients to ensure or improve some qualitative characteristics of foods, such as wine and ice cream, acting as antioxidants and antimicrobials, and/or increasing their nutritional value, being a source of fiber.

### 3.1. Substitution of Sulfur Dioxide in Winemaking

Sulfur dioxide (SO_2_) is one of the most used additives in the winemaking industry, due to its antioxidant and antimicrobial activity. In fact, it is used to control unwanted microorganisms and enzymatic activities during winemaking, controlling oxidative processes and unwanted fermentations [56,78]. However, its excessive use is associated with adverse effects on human health related to dermatitis, urticaria, bronchoconstriction, and anaphylaxis; and defects related to organoleptic alterations, such as neutralization of aromatic compounds and the appearance of defects in wine, increasing unpleasant flavors and smells, and increasing turbidity [60,78]. For this reason, there is great interest in replacing or limiting the levels of SO_2_ added in wines, and among the most used methods are high pressure, ultrasound, and pulsed electric fields, which exert only the antimicrobial effect but not the antioxidant activity [56,60]. Therefore, the addition of natural compounds, including lysozyme, hydroxytyrosol, oenological tannins, and plant extracts has been tested [55,79]. In recent years, there is a growing tendency to use wine by-products, such as grape stalks and vine shoots—due to the presence of phenolic acids (caffeic acid, gallic and p-coumaric acid), flavonoids (catechin, epicatechin, and luteolin), and stilbenes (trans-resveratrol and its trans-ε-viniferin dimer)—to replace SO_2_, thus acting as natural antioxidants and stabilizers of wine, contrasting the formation of aggregates between proteins and SO_2_, the cause of opacity and instability [80,81,82,83,84,85,86].

#### 3.1.1. Stilbenes

Grape stalks extract rich in stilbenes was added by Ruiz-Moreno et al. [55] in amounts of 50 and 80 mg/L in a model wine in order to both reduce the SO_2_ in winemaking and study the effect on the aroma. The results showed a higher antioxidant effect, a lower antimicrobial effect against *Saccharomyces cerevisiae* and *Hanseniaspora uvarum*, and a higher antimicrobial effect again *Candida stellata* and *Botryotinia fuckeliana* compared to SO_2_. The olfactometric profile evaluated by the gas chromatography–olfactometry technique, instead, was similar to the typical one of wines. Raposo et al. [56], instead, used a commercial extract of stilbenes obtained from vine shoots in order to preserve the quality of a red wine, studying the effectiveness at bottling and the storage in bottle for twelve months. The addition, in an amount of 86 mg/L of wine (25 mg/L of total stilbenes), increased the intensity and purity of the color and led to a high score at sensory analysis, evaluated at bottling. Moreover, the use of commercial stilbenes improved the aroma of wines and preserved volatile compounds, showing more notes of black and mature fruit. However, after aging, the extract did not allow the quality to be preserved compared to wines treated with SO_2_. Based on these results, in subsequent research the authors evaluated the optimal dose to increase the qualitative parameters and the sensory attributes, at two concentrations of added extract, namely 175 mg/L of wine (50 mg/L of the total stilbene content) and 430 mg/L (100 mg/L of the total stilbene content), during twelve months of storage in bottle. The obtained results were similar to the SO_2_-treated wines, but with differences in phenolic composition and color intensity. The wines obtained, in fact, had high concentrations of vinyl-pyranoanthocyanins and B-type vitisins, two stable color compounds resistant to oxidation, and a lower concentration of free anthocyanins. Moreover, the addition of extract led to the modification of sensory attributes related to black fruit, caramel, and woody when 430 mg/L of extract was used, which also led to a decrease of scores related to global quality. The same quantities tested by Ruiz-Moreno et al. [58] preserved phenolic compounds and improved the chromatic characteristics of wines and did not adversely affect the aromatic composition and sensory properties, showing high concentrations of β-damascenone, an odorant associated with descriptors such as fruity, honey, and baked apple, and slight astringent notes. In the same way, Cruz et al. [59] reported that quantities of 138 mg/L of commercial stilbenes, added in white wines at bottling, positively influenced the sensorial characteristics, identifying white fruit attributes and higher aromatic intensity, without any defects, despite slight negative effects on color, until six months of storage.

#### 3.1.2. Phenolic Acids, Flavanols, and Tannins

The effect of the use of extracts from grape seeds and stalks alone or in combination with colloidal silver complex was evaluated by assaying the antioxidant and antimicrobial activity and determining color, phenolic and volatile composition, and sensorial profile of white wines [60]. Wines treated with extracts of grape stalks rich in hydroxycinnamic acids, catechin, epicatechin, and procyanidin B1 and B2 showed no differences in terms of antioxidant activity and total yeasts, and lactic and acetic bacteria counts with respect to the wine treated with SO_2_. The chromatic parameters were increased by the addition of 0.5 g/L of grape stalk extract, and 0.5 g/L of grape stalk extract and 1 g/L of colloidal silver complex; while from a microbiological point of view, no differences were found compared to control wines (treated with SO_2_). On the other hand, the phenolic and volatile composition of wines was modified using extracts, showing higher quantities of flavonols and ethyl esters and lower concentrations of acetaldehyde. In addition, the grape stalk extracts affected sensory attributes, increasing the scores related to fruity and floral. The effect of addition of grape stalks on the color and phenolic content in red wine was evaluated also by Pascual et al. [61]. The use of grape stalks increased the concentration of catechins, gallocatechins, and proanthocyanidins but led to a decrease in the intensity of color and an increase in the astringency and bitterness of wine. Other authors also reported that the volatile and phenolic composition may also be influenced by the addition of vine shoots. Cebrián-Tarancón et al. [62,63], in fact, studied the chemical composition of model [62] and real wines [63] after the use of toasted and not toasted vine shoots, in different particle sizes and at different maceration times. Positive effects were reported especially after 35 days of maceration and using toasted vine shoots at the concentration of 12 g/L, obtaining wines with high levels of vanillin, trans caftaric acid, caffeic acid, and trans resveratrol. Overall, the ability to preserve the quality of wines and to contribute to the sustainability of the wine chain allows these by-products to be used as an alternative to the classic wine additives in order to improve the phenolic profile despite slight negative effects on color and astringency of wines.

### 3.2. Substitution of Bentonite in Winemaking

The possibility of replacing bentonite with dried and ground grape stalks, was considered by Kosińska-Cagnazzo et al. [64], with the aim of removing the unstable proteins that cause turbidity in wines. The addition of 10 mg/mL in a wine model precipitated all the proteins, preserved the total polyphenols content, but caused a change in color of wine at high doses. The quantity of proteins remaining in wine, in fact, was inversely proportional to the addition of grape stalks, due to the interactions between proteins and polyphenols that promote the formation of precipitates. This could be exploited, for example, in white wine rich in insoluble proteins that precipitate slowly and poor in tannins useful for the initial protein precipitation.

### 3.3. Production of High-Added Value Foods

As a result of the growing global interest in the relationship between nutrition and health, recent studies focused on the development of innovative food products [70,87]. Some researchers have analyzed the possibility of integrating agro-food by-products to produce foods improved by a nutritional point of view.

For this purpose, Borges et al. [70] added wine lees to produce cereal bars. Wine lees represent a precious by-product linked to the presence of insoluble carbohydrates (from cellulosic and hemicellulosic fractions), phenolic compounds, and lignin, but especially proteins, which make wine lees nutritionally very rich [70,73].

To increase the nutritional value of the bars, the authors used wine lees subjected to an autolysis process to break the yeast cell walls to facilitate the release of accumulated proteins inside. The results showed that the addition of this by-product at 2.5 and 5% allows cereal bars to be obtained that contribute to the recommended daily protein intake; from a sensory point of view, instead, the addition of wine lees made the sample darker but acceptable to the taste by consumers without finding substantial differences compared to unfortified cereal bars.

The impact of adding wine lees to ice cream production was assessed by Hwang et al. [71] through the addition of 50, 100, and 150 g/kg of wine lees homogenized with distilled water. The results showed that the addition of 50 g/kg of wine lees improved the characteristics of the ice cream, leading to a decrease in specific gravity, pH, firmness, lightness, and freezable water amount and a concomitant increase in viscosity, melting rate, yellowness, and fat destabilization. In addition, the fortified product showed higher antioxidant activity, confirming that the compounds present in wine lees were stable during the production process. However, the addition at higher amounts determined a negative effect related to the increase of the particle size of fat globules and overrun (the amount of air incorporated during the batch freezing). Pundhir et al. [72], instead, added 35 g/kg of wine lees to produce a fortified ice cream with low sugar content. The product showed an increased phenolic content with improved physical, functional, and rheological properties, while a decrease in pH, specific weight, and overrun due to increased viscosity and an increase in the destabilization index of fats were also observed. The sensory analysis showed a higher color intensity and overall pleasantness than control without extract, as also found by Sharma et al. [73].

Microbiological aspects related to the addition of wine lees in ice cream were investigated by Ayar et al. [74], who evaluated the vitality of *Lactobacillus acidophilus* (ATCC 4357D-5) and *Bifidobacterium animalis* subsp. *lactis* (ATCC 27536) after 1, 15, 30, and 60 days of storage. The presence of dietary fiber from wine lees had a positive effect on the survival of *L. acidophilus* during storage, but slightly decreased the count of *B. animalis* subsp. *lactis* whose decrease was less than 1.0 log colony-forming unit (CFU)/g, however, showing concentrations greater than 6 log CFU/g, such as to define ice cream as a probiotic (European Communities (EC) Regulation No 1924/2006). Moreover, the addition of wine lees improved the physico-chemical and sensory characteristics, as described in previous studies.

Wine lees have also been tested as preservatives to replace the most commonly used additives in meat products. This kind of food tends to oxidize rapidly during storage, so Alaracon et al. [75] estimated the effect of adding wine lees at 2.5 and 5% in deer burgers packaged in modified atmosphere at 4 °C. The fortification countered lipid and protein oxidation due to increased phenolic content and antioxidant activity. Moreover, the addition of wine lees had an antimicrobial effect against psychotrophic aerobic bacteria and modified the organoleptic characteristics of the products. After sensorial analysis, in fact, the hamburgers had attributes like wine and bakery notes, considered pleasant at low intensity, due to the increase of benzene compounds, esters, and acids present initially in wine lees. The addition of lees preserved the typical color of meat, with a low decrease in values of redness (a*) compared to the control samples.

### 3.4. Improvement of Alcoholic Beverage Quality

Wine quality is an essential element for the marketability of the product, which depends on both oenological and cultivation techniques. In recent years, attention to sustainability of production in general and viticulture has become an increasingly topical requirement. For these reasons, researchers paid attention to finding alternatives to traditional fertilizers. Currently, aqueous oak extracts are used, because the compounds present are assimilated by the grape and transmitted in the wine, influencing both quality and composition [88]. Sánchez-Gómez et al. [65] studied the chemical composition of aqueous extracts of toasted and untoasted vine shoots, highlighting an interesting composition in terms of phenolic, volatile, and mineral compounds, that if assimilated by plants by foliar applications, could improve the amino-acid, volatile, and phenolic composition of wines obtained.

The aqueous formulations were utilized for foliar treatments, on the seventh day post-veraison, comparing the results with the effect of the treatment with water and adjuvants. The use of this innovative fertilizer allowed an improvement in the volatile composition of wines to be obtained, probably due to the increasing amino acid content. Some amino acids, in fact, such as phenylalanine, leucine, isoleucine, and valine are precursors of volatile compounds important in wine [89,90]. In other studies, Sánchez-Gómez et al. [32,66] tested the effect of two different extracts of vine shoots, one deriving from untoasted and the other from toasted vine shoots, by applying them on the leaves at veraison. Both extracts improved the classic parameters such as pH, total and volatile acidity, and color and positively influenced phenolic and volatile composition of the wine. In addition, the foliar treatment increased grape yield, expressed as kg/plant, and induced a decrease in the alcohol content of the wine. In fact, treated plants had grapes with a lower °Brix than control ones, probably due to a stress reaction induced by the use of extracts, which led to decrease of the photosynthesis process and consequently of sugar concentration. Sánchez-Gómez et al. [32] also characterized the floral and fruity descriptors of wines, finding higher spicy notes due to the presence of compounds such as norisoprenoids (β-damascenone), vanillin derivatives, guaiacol, and syringol, as well as an increase in the content of stilbenes, especially when the vine shoots were toasted. In another study, Sánchez-Gómez et al. [67] carried out foliar applications with vine shoot extracts in order to evaluate the concentration of phenolic compounds and glutathione in the must and wine, as well as the color of white wines, which very often tends to be modified due to oxidation. In this context, glutathione plays a key role in giving origins to compounds capable of inhibiting the browning of wines. The results showed that the foliar application with vine shoot extracts did not influence the concentration of glutathione compared to other treatments that otherwise decrease it, obtaining an overall improvement in the color of the wines produced.

The quality of the wine was also improved by Miljić et al. [68] by adding grape seeds and stalks during the vinification in red. The presence of stalks increased the phenolic content and antioxidant activity compared to control wines; however, the experimental wine showed pronounced herbal notes, which reduced consumer acceptability when 50% of grape stalks were used, unlike the addition of 25%, which improved sensory characteristics and increased acceptability.

An innovative liqueur obtained from red fruit and fortified with grape stalks, was produced by Barros et al. [69], who used this by-product as a source of polyphenols. The liqueur was obtained by adding 50 g of grape stalk powder in marc grape spirits and sugar, subjected to a maceration phase, and analyzed after 90 and 180 days. The product obtained had a higher antioxidant activity due to the presence of bioactive compounds (ortho-diphenols, flavanols, flavonols, and anthocyanins) compared to a liqueur obtained without the addition of grape stalks. The highest value of antioxidant activity was achieved after 90 days of maceration, also showing a change in color parameters. The value of lightness (L*) decreased due to the precipitation of particles of plant material that increased turbidity; the values of redness (a*) and yellowness (b*) instead increased, obtaining an innovative liqueur with an attractive color.

### 3.5. Inhibition of Food Pathogens

The inhibiting effect of grape stalk polyphenols was assayed against the growth of some food pathogens, such as *Listeria monocytogenes*, *Staphylococcus aureus*, *Salmonella enterica* subsp. *enterica* serovar Typhimurium, and *Escherichia coli O157: H7*. As an alternative to synthetic antimicrobials, grape stalk extracts were applied on samples of fresh leafy vegetables (lettuce and spinach). The results related to antimicrobial activity of extract showed a high inhibition efficacy at a concentration of 25 g/L, especially against *E. coli* O157: H7 and *Salmonella enterica*, showing a reduction of about 2 logarithmic units [76]

In a subsequent study, Vázquez-Armenta et al. [77] evaluated the effect of grape stalks extracts, compared to synthetic disinfectants, on motility, surface energy, and adhesion of *Listeria monocytogenes* on food contact surfaces, such as stainless steel and polypropylene. The results obtained after the use of 18 and 20 mg/mL of grape stalk extracts on both surfaces showed a greater reduction in pathogen adhesion than the control; the presence of grape stalk extracts probably induced the synthesis of exopolysaccharides, which is responsible for the inhibition of motility, adhesion, and biofilm formation. In addition, a greater impact of phenolic compounds such as ferulic, gallic, and caffeic acid, compared to flavonoids such as catechin and rutin, on the inhibition of *Listeria monocytogenes*, was shown. The in vitro antimicrobial activity of grape stalks after 64 days of storage against positive and negative Gram bacteria was studied by Gouvinhas et al. [49]. In particular, antimicrobial activity was tested against *Listeria monocytogenes*, *Staphylococcus aureus*, *Enterococcus faecalis*, *Pseudomonas aeruginosa*, *Escherichia coli*, and *Klebsiella pneumoniae*, which are known as foodborne pathogens. The high phenolic content, in particular ortho-diphenols and flavonoids, allows, therefore, the extracts to be used as antimicrobials for the high inhibitory power shown, in some cases, to be greater than commercial antimicrobials.

## 4. Food Packaging Formulations

In recent years, the massive use of plastic materials has inevitably led to an increase in issues related to environmental pollution and the disposal of these materials [91]. To tackle this problem, interest in sustainable packaging made with biopolymers from renewable agro-industrial sources, widely available and cheap, is growing [92,93,94]. In Table 2 are reported the possible applications of vine and winery by-products as fillers for food packaging.

In order to find a substitute for expanded polystyrene (EPS) in the trays, Engel et al. [91] added 18.4% (*w*/*w*) of grape stalks, known for their richness in lignocellulosic fibers, and glycerol (as plasticizer) to the polymer fraction (cassava starch-based foams). This increased the tensile strength and elasticity of starch-based foams through the interfacial interaction that occurs between the matrix and the fibers, obtaining foams with good mechanical properties, low density, and increased moisture resistance and hydrophobicity. In a subsequent study, the same authors carried out tests of applicability on low moisture foods (cake) of biodegradability of polymer matrix added with 7% (*w*/*w*) of grape stalks [95]. The applicability tests on foods, compared to the use of expanded polystyrene (control), highlighted the appearance of deformations in correspondence of the contact points between cake and packaging and the increase in moisture in the first days of storage; however, after nine days of storage the samples showed no significant difference in moisture content. These results showed a possible use of the innovative packaging for foods with low moisture content as an alternative to expanded polystyrene materials for short-term storage. The study on biodegradability, instead, showed that the amorphous structure of the innovative packaging contributes to rapid biodegradation, which is useful for reducing environmental problems and for the disposal of waste and by-products.

El Achaby et al. [96] studied the possibility of producing cellulose nanocrystals (CNCs) from vine shoots composed of about 70% holocellulose (cellulose and hemicellulose) and 20% lignin. After extraction, the CNCs were added in amounts of 1, 3, 5, and 8% (*w*/*w*) as reinforcements for the production of materials with carboxymethyl cellulose as a biopolymer matrix. The mechanical properties of the obtained material highlighted a final structure with a high crystallinity (82%) and thermal stability, and good colloidal stability in water, thus obtaining a biopolymer with good properties of elasticity and tensile strength.

Vine shoots were further applied as fillers of a matrix of poly(3-hydroxybutyrate-3-hydroxy-valerate) (PHBV) by adding 20% (*w*/*w*) of grounded vine shoots [97]. As a result, an acceleration of the biodegradation kinetics of the material in the soil, as well as packaging with good structure and morphology properties, were found. Nanni et al. [98,99], in two subsequent studies, tested the use of wine lees as fillers at 10, 20, and 40 phr (parts for hundred parts of polymer) in biodegradable biopolymers, such as poly(3-hydroxybutyrate-cohydroxyhexanoate) (PHBH), poly(3-hydroxybutyrate-cohydroxyvalerate) (PHBV), and polybutylene succinate (PBS). The obtained results on tensile and creep tests, scanning electron microscopy, and differential scanning calorimetry seemed to be promising. In fact, the addition of wine lees increased the Young’s modulus without decreasing the tensile strength due to the good adhesion between wine lees particles and the matrix.

## 5. Conclusions

The different researches examined in this review show a growing interest in vine and winery by-products, which are not managed as wastes but as a source of functional compounds to be exploited in the production of innovative food and packaging. The presence of bioactive compounds, such as polyphenols and dietary or lignocellulosic fiber, allows these by-products or their extracts to be used as (i) antioxidant or stabilizing agents, replacing the most used oenological additives; (ii) plant bio-stimulants, obtaining grapes and wines with high quality characteristics; (iii) antimicrobial agents against food pathogens; (iv) phenolic content improvement; and (v) alternatives to petrochemical polymers, ensuring the environmental sustainability. Their valorization, therefore, allows one to give a second life to the winery by-products, contributing at the same time to the reduction of production costs and residual quantity. In fact, national legislation, international regulatory frameworks, and directives concerning waste management indicate waste prevention/minimization and by-product valorization as key strategies for the effective management system and sustainability of the food industry.

## Figures and Tables

**Table 1:** Applications of vine shoots, grape stalks, and wine lees or their extracts in food and beverages.

Aims	Compounds	Treatment	Source	Result	References
SO_2_ substitution in wine	Stilbenes	Hydroalcoholic extraction	GS	Increased antioxidant and antimicrobial activity in wine model.	[55]
			VS	Improvement of color intensity and sensory characteristics until 12 months of storage.	[56]
			VS	Improvement of color intensity, phenolic compounds, and quality of wine.	[57]
			VS	Stabilization of anthocyanins and improvement of the chromatic properties of wine, polyphenol stabilization and aromatic profile of wine.	[58]
			VS	Change of the aromatic profile and color of white wine.	[59]
	Phenolic acids, flavanols and tannins	Aqueous extraction	GS	Increase of antioxidant and antimicrobial activity in treated wines; increase attributes related to floral and fruity aroma.	[60]
		-	GS	Increase of phenolic compounds, astringency, and bitterness and decrease of color intensity.	[61]
		Toasting and untoasting chips and granules	VS	Positive modification of flavor of model wine.	[62]
			VS	Improvement of chemical composition of wines in terms of phenolic substances and antioxidants.	[63]
Bentonite substitution	Phenolic acids, flavanols and tannins	Drying and milling	GS	Removal of unstable proteins for protein precipitation.	[64]
Improvement of wine quality	Phenolic compounds	Aqueous extraction	VS	Increase of content of amino acids in must, and volatile composition of the wines.	[65]
			VS	Improvement of wine quality in terms of pH, total acidity, volatile acidity, intensity of color, aroma, and volatile and phenolic compounds.	[66]
			VS	Improvement of white wine quality in terms of aromatic profile and phenolic compounds.	[32]
			VS	Increase of phenolic compounds and preservation of glutathione content.	[67]
		Raw sample	GS	Improvement of antioxidant activity and phenolic content of wines and increase of herbal notes in taste and flavor.	[68]
Production of grape stem-based liqueur	Phenolic compounds	Crushing	GS	Improvement of phenolic compound, antioxidant activity, and intensity of color of liqueur after 90 days of maceration.	[69]
Production of fortified cereal bar	Protein	Drying, crushing, and autolysis	WL	Improvement of protein content of cereal bars; small difference in color and taste.	[70]
Production of high-added value ice cream	Phenolic compounds and dietary fiber	Homogenization with water	WL	Improved ice cream structure and properties; increased antioxidant and inhibitory effect towards the oxidation of human erythrocyte membranes.	[71]
		Freeze-drying and homogenization with water	WL	Increase of phenolic content and improvement of physical, functional, and rheological properties.	[72]
			WL	Improvement of the physical–chemical, rheological, and sensory properties of ice cream.	[73]
		-	WL	Production of ice cream with physical–chemical and sensory properties comparable to control ice cream; increase of survival rate of *Lactobacillus acidophilus* during 60 days of storage.	[74]
Synthetic additives substitution in hamburger	Phenolic compounds	Freeze-drying	WL	Increase of antioxidant and antimicrobic activity and phenolic compounds in burger.	[75]
Inhibition of food pathogens	Phenolic compounds	Freeze-drying and hydroalcoholic extraction	GS	Inhibition of *Listeria monocytogenes*, *Staphylococcus aureus*, *Salmonella enterica* subsp. *enterica* serovar Typhimurium, and *Escherichia coli* O157: H7 in lettuce and spinach.	[76]
			GS	Reduction of adhesion potential and *Listeria monocytogenes* motility on food contact surfaces (steel and polypropylene).	[77]
			GS	High antimicrobial activity, after 64 days of storage, against gram+ and gram− bacteria.	[49]

GS, grape stalks; VS, vine shoots; WL, wine lees.

**Table 2:** Applications of wine by-products as filler to improve food packaging.

Matrix	Compounds	Treatments	Source	Result	References
Cassava starch-based foams	Lignocellulosic insoluble fibers	Drying and crushing	GS	Foams with good mechanical properties and increased moisture resistance.	[91]
Cassava starch-based foams	Lignocellulosic insoluble fibers	Drying and crushing	GS	Good mechanical and biodegradable packaging properties and low moisture resistance.	[95]
Carboxymethyl cellulose	Cellulose	Alkali, bleaching, and acid hydrolysis treatments	VS	Increase of elastic modulus and tensile strength, crystallinity, and high thermal stability of biopolymer incorporated with cellulose nanocrystals from vine shoots.	[96]
PHBV	Lignocellulosic insoluble fibers	Drying and milling	VS	Complete biodegradation with fillers consisting of vine shoots are exhausted.	[97]
PHBH and PHBV	Inorganic fractions	Drying and grounding	WL	Production of biopolymers, such as PHBH and PHBV, with good thermal, mechanical, rheological, and morphological characteristics.	[98]
PBS	Inorganic fractions	Air-cooling and grounding	WL	Production of biopolymers, such as PBS, with good thermal, mechanical, rheological, and morphological characteristics.	[99]

PHBV, poly(3-hydroxybutyrate-3-hydroxy-valerate); PHBH, poly(3-hydroxybutyrate-cohydroxyhexanoate); PBS, polybutylene succinate; GS, grape stalks; VS, vine shoots; WL, wine lees.
